# Civil Surgeons and the War

**Published:** 1900-12-01

**Authors:** 


### The Hospital

A Journal of

The Medical Sciences and Hospital Administration.

Vol. XXIX.—No. 740.] Edited by Sir Henry Burdett, K.C.B., and Solomon C. Smith, M.D., M.R.C.P. [December 1, 1900.

### Civil Surgeons and the War.

The approaching return from South Africa of the Langman and of the Irish Hospitals, both of them furnished, equipped, and controlled by civilian liberality and by civilian surgeons, and both of them having done excellent service to the sick and wounded, while on the one hand a gratifying evidence that, in the opinion of the military authorities, the real brunt of the struggle is over, and the remaining business of suppressing bands of mere marauders is a comparatively trivial, although probably a troublesome task, certainly calls upon the country, on the other, to recognise, in some effective way, the admirable character of the work which these hospitals and others have carried through. The civilian surgeons who first returned, Sir William MacCormac, Mr. Treves, and Mr. Dent, and who were therefore the first to lay before the profession and the public some account of their adventures, and of the conditions against which they had to contend, not unnaturally may be said to have carried off some of the cream of popular applause; but we trust that this circumstance will not be permitted to detract from the full recognition of what is due to others who have been somewhat less acclaimed by the Press, and who have laboured assiduously and unpretendingly from the beginning to the end of the strife. It is certain that the medical organisation of the Army would have hopelessly broken down if it had not been for civilian assistance; and the fact that it would have done so does not, in our estimation, furnish any considerable addition to the indictment against the War Office which is said to be in preparation in high quarters. A great war comes only once in thirty or forty years, and politicians do not seem yet to have learned that its coming is inevitable, and should be provided against by what has been happily called "an intelligent anticipation of events." It is too often assumed that each great war will be the last; an assumption which rests upon a cheerful ignoring alike of the lessons of history and of the facts and tendencies of human nature. The War Office, in contenting itself with the medical organisation necessary for an army in time of peace, and in neglecting all provision for its rapid expansion on the outbreak of war, has not been much more blamable than the community by which the neglect in question has at least been tacitly sanctioned. How much may be done by even a limited amount of preparation has been admirably shown by the Order of St. John, which, as a result of its regular ambulance work, was able at once to supply fifteen hundred men—all Volunteers—who had been sufficiently trained in rendering "first aid" to the wounded to be able to take their place as useful workers in the field. Would it not be possible, under the auspices either of the Order or of the War Office itself, to build up in time of peace a complete medical organisation, to be supplementary to that of the Army in time of war, and to be in a state of constant preparedness for the duties which war would call upon it to undertake? We believe that the medical profession would respond cordially to such a demand, and that its members would be abundantly ready to enrol themselves as Volunteers for field service under any fair conditions of a kind calculated to show that their patriotism would be estimated at its true value by the public.

By no means the least useful part of the good work done by our doctors, whether civil or military, during the war has been the cordial extension of their services to the wounded of the enemy; an extension which has not been merely perfunctory, or grudgingly performed, but has been of a character to exercise a very important influence upon the future of the two races, which will, we hope, soon be living together in amity. Foolish people, or malignant ones endeavouring to conceal their malignity under a cloak of foolishness, are never weary of talking and writing about the evil passions which the war will engender, and the hatreds which will follow in its train. They, like the peace optimists, are either ignorant or forgetful of the teachings of history. Such hatreds, as results of war, seldom descend to the civilian population, and seldom survive the actual combatants. They do not survive personal experience of good and kindly offices. The English soldiers in 1814 were received in France as saviours and benefactors by a population weary of the ravages of war, and the whole audience at the Paris Opera House rose to welcome the Duke of Wellington when he entered his box. On one of the few occasions when Englishmen and Boers came to close quarters the Boers defended a kopje which the English carried by assault. An English soldier, shot by a Boer within a few steps of the goal, and badly wounded, was still able to reach his assailant with the bayonet, and the two men fell together. They were picked up by the same ambulance, taken to the same hospital, and put into adjoining beds. They became warm friends during their sickness and convalescence, played draughts, shared tobacco, exchanged confidences about their respective families and affairs, and formed projects of co-operation after the war, if the Englishman could arrange to stay in, or to return to, the country. As they shook hands before parting, with many promises to meet again, the Englishman was heard to say, "I say, old man, *what a good thing it was we met!*"

